# Frailty and health services use among Quebec seniors with non-hip fractures: a population-based study using adminsitrative databases

**DOI:** 10.1186/s12913-019-3865-z

**Published:** 2019-01-25

**Authors:** Vanessa Fillion, Marie-Josée Sirois, Philippe Gamache, Jason Robert Guertin, Suzanne N. Morin, Sonia Jean

**Affiliations:** 1 Centre d’Excellence sur le Vieillissement de Québec (CEVQ), Québec, Canada; 2 Centre de recherche du CHU de Québec, Québec, Canada; 3 The Canadian Emergency Team Initiative (CETI), Québec, Canada; 4 Bureau d’information et d’études en santé des populations, Institut national de santé publique du Québec (INSPQ), 945, avenue Wolfe, Québec, QC G1V 5B3 Canada; 5 Université Laval, Québec, Canada; 6 Université McGill, Québec, Canada; 7 Hôpital de l’Enfant-Jésus, 1401 18e rue, H-602, Québec, QC G1J 1Z4 Canada; 8 Hôpital Saint-Sacrement, 1050 Chemin Sainte-Foy, Bureau J0-01, Québec, QC G1S 4L8 Canada; 9 Montreal General Hospital, 1650 Cedar Avenue, Room B2.118, Montréal, QC H3G 1A4 Canada

**Keywords:** Frailty, Elderly, Fracture, Health administrative database

## Abstract

**Background:**

The number of frail elderly will increase as the world population ageing accelerates. Since frail elders are at risk of falls, hospitalizations and disabilities, they will require more health care and services. To assess frailty prevalence using health administrative databases, to examine the association between frailty and the use of medical services and to measure the excess use of health services following a non-hip fracture across frailty levels among community-dwelling seniors.

**Methods:**

A population-based cohort study was built from the Quebec Integrated Chronic Disease Surveillance System, including men and women ≥65 years old, non-institutionalized in the pre-fracture year. Frailty was measured using the Elders Risk Assessment (ERA) index. Multivariate Generalized Estimating Equation models were used to examine the relationship between frailty levels and health services while adjusting for covariates. The excess numbers of visits to Emergency Departments (ED) and to Primary Care Practitioners (PCP) as well as hospitalizations were also estimated.

**Results:**

The cohort included 178,304 fractures. There were 13.6 and 5.2% frail and robust seniors, respectively. In the post-fracture year, the risks of ED visits, PCP visits and hospitalizations, were significantly higher in frail vs. non-frail seniors: adjusted relative risk (RR) = 2.69 [95% CI: 2.50–2.90] for ED visits, RR = 1.28 [95% CI: 1.23–1.32] for PCP visits and RR = 2.34 [95% CI: 2.14–2.55] for hospitalizations.

**Conclusion:**

Our results suggest that it is possible to characterize seniors’ frailty status at a population level using health administrative databases. Furthermore, this study shows that non-institutionalized frail seniors require more health services after an incident fracture. Screening for frailty in seniors should be part of clinical management in order to identify those at a higher risk of needing health services.

**Electronic supplementary material:**

The online version of this article (10.1186/s12913-019-3865-z) contains supplementary material, which is available to authorized users.

## Background

In parallel with an aging population, the number of frail elderly is increasing. Worldwide reported prevalence of frailty vary greatly (from 4 to 59%), with an overall weighted estimate of around 13% [1], thereby imposing an important burden on the planning and delivery of health and social services [2].

Frailty is a central concept in geriatric medicine, and is defined as a generalized reduction of homeostatic reserves in multiple physiological systems leading to a state of vulnerability, which increases the risk of adverse outcomes such as delirium, falls and disabilities [2]. As a consequence, frailty is associated to disproportionate changes in health status even when following relatively minor stressor events [2]. For instance, it has been demonstrated that, compared to robust seniors, frail individuals have a higher risk of falls [2] and a higher risk of sustaining low-trauma fractures [3, 4]. Moreover, frail community-dwelling seniors with minor fractures have been shown to experience increased physical, emotional and social disabilities in the 6 months following such minor trauma, when compared to non-frail seniors [5]. For those who are hospitalized after a fracture, seniors who are frail are at an increased risk of being discharged to a long-term care institution [5].

The complex frailty mechanisms are also influenced by a large range factors (genetic, biological, environmental, social, etc.), [2, 6–9] and as a consequence, older patients are a heterogeneous group in which the expression of frailty may involve co-morbidities as well as multidimensional functional losses (physical, cognitive, psychological, social) [8, 10–13] that are likely to require a broad array of health care and services [2, 14]. Unfortunately, studies examining the relationships between frailty and use of health services are scarce. In the “*Belgian health interview survey*” [15] cross-sectional cohorts of seniors living at home, increased frailty was found to be independently associated with increased self-reported use of primary care practitioners (PCP), nursing, home help services and hospitalizations. In the “*Survey of Health Ageing and Retirement in Europe*” (SHARE cohorts) [16], frail seniors showed increased utilization of primary and hospital care prior to onset of frailty-related disabilities. Furthermore, in the “*Concord Health and Ageing in Men Project*” (CHAMP cohort), *Rochat* et al. found that increased frailty was strongly associated with increased use of health and community services in community-dwelling older men [17]. Finally, in the Canadian Emergency Team Initiative (CETI) cohorts in which a 11.3% frailty prevalence was found among community-living seniors discharged back home after consulting emergency departments (ED) for minor injuries such as non-hip minor fractures [18], increased clinically measured frailty was associated with increased self-reported ED visits, hospitalizations, physiotherapist and home care services use up to 6 months post-injuries [19].

Cohort studies and clinical trials are currently used to study information on the identification of frail seniors (measured generally using clinical indices or scales) and on their health resources needs and use [20–23]. Among those, the *Fried phenotype of physical frailty* [20] and the *Canadian study of health and aging- clinical frailty scale* [23], are some of the most commonly generated frailty measures in both research and geriatric practice. Even if they are simple, these measures are not currently recorded in clinical settings. Hence, they are generally not included in administrative databases available for health services planning and delivery, surveillance of health status population or various researches. However, given that the number of frail elderly is increasing, methodologies to identify frail seniors within administrative data (patient and population levels) are current surveillance priorities [24]. Moreover, such frailty identification should capture the current views on frailty and include more than its physical dimensions, also integrating its psychological, cognitive and social components, in order to reflect the multisystem and multidimensional impairments and consequences that are intrinsic to this concept [8, 10–13]. Some authors even consider attempting to account for risk factors of frailty such as social support, which may mediate frailty and its consequences [13] such as health care use.

In that context, *Crane* et al. conducted a retrospective cohort study using an electronic administrative database which included 12,650 community-dwelling adults assigned to a primary care internal medicine provider in Rochester, Minnesota [25]. They developed and validated the multidimensional Elders Risk Assessment (ERA) index to prospectively stratify frail community-dwelling seniors for the risk of total number of emergency room (ED) visits and hospitalizations over 2 years. *Soong* et al. also conducted a retrospective cohort study using administrative data on 2,099,252 seniors with ED admission to National Health Service in the UK [24]. They included in their analysis patient demographics, frailty syndromes, previous service use, inpatient mortality, 30-day ED readmission and increase functional dependence at discharge [24]. These recent studies tend to indicate that surveillance data may contribute to identifying population subgroups affected by frailty and help to determine their health care needs.

In the current study, using the Quebec health administrative databases, we sought to parallel the above mentioned CETI cohort of seniors with minor fractures at a population level, in order to 1) assess the prevalence of frailty among community-dwelling seniors with a minor fracture in the province of Quebec using the ERA index, 2) examine the association between frailty and the use of medical services (ED visits, PCP visits, hospitalizations) in the year following the minor fracture, and 3) measure the excess use of health services following the fracture across frailty levels.

## Methods

### Study design and data source

This study is a population-based retrospective cohort built from the Quebec Integrated Chronic Disease Surveillance System (QICDSS), an innovative chronic disease surveillance system linking five healthcare administrative databases covering the health services offered to all residents in the province of Quebec, Canada [26]. These provincial linked databases include: health insurance registry (FIPA), the hospital discharges abstracts (Med-Echo), the vital statistics & deaths, the physician-billing claims (PCD), and the pharmaceutical services. For 2015–2016 the QICDSS contained information on 8,222,852 Quebecers. The creation of the QICDSS and data access both meet strict requirements of security and privacy. Its creation was approved by the government agencies in legal possession of the databases, the Public Health Ethics Board and by the “Commission d’accès à l’information” [26], and consequently, ethics approval and participant consent was not necessary for this study.

This study uses three specific data sources from the QICDSS: 1) FIPA, which includes insurance eligibility and demographic information; 2) Med-Echo, containing information on inpatient discharges from all Quebec hospitals that provide general or specialized care (length of stay, primary and secondary diagnoses, all hospital care provided, destination at discharge, etc.) [26]. Diagnoses are coded using the International Classification of Diseases, 9th Revision, Clinical Modification (ICD-9-CM) before April 1, 2006 (16 diagnostic codes), and the ICD, 10th Revision, Canada (ICD − 10-CA) thereafter (26 diagnostic codes). Therapeutic interventions are recorded using the Canadian Classification of Diagnostic, Therapeutic, and Surgical procedures (CCP with ICD-9-CM) and the Canadian Classification of Interventions (CCI with ICD-10-CM); 3) PCD containing data related to fee-for-service billings, that is the payment claims that health professionals submit to the Quebec Universal Health Insurance Board (Régie de l’assurance maladie du Québec - RAMQ) [26]. Each record includes information related to physician reimbursement (billing codes for the clinical services, dates and locations of the clinical services provided, and a ICD-9-CM diagnosis code) [26].

### Participants

**Fig. 1:**
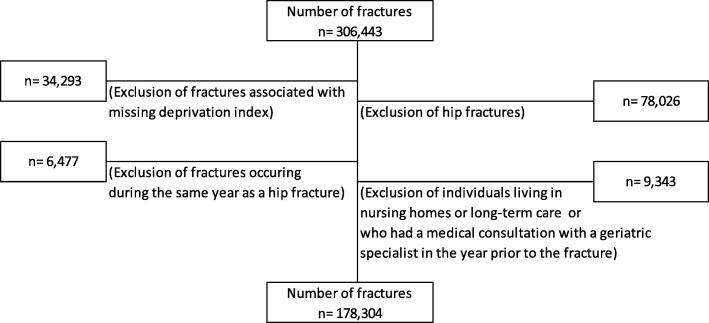
Flow chart of the study

From the QICDSS databases and in order to parallel the CETI cohorts that excluded hip fractures because they require hospitalisation, all non-hip fractures among community-dwelling men and women aged 65 and over occurring between January 1, 1997 and December 31, 2014 were included and identified with a previously validated algorithm [27]. Fractures associated to patients having missing material and social deprivation index (see covariates section for description of this index) were excluded due to a lack of social components necessary to complete the ERA index. Fractures occurring in the same year as a previous hip fracture were excluded since resource use related to each fracture cannot be distinguished. All fractures sustained by patients living in nursing homes and long-term care, or who had a medical consultation with geriatric specialists in the year before their fracture, were excluded from the analyses. The final sample includes 178,304 seniors with non-hip fractures. See Fig. 1 for the study flowchart.

### Measures

#### Frailty

Frailty status at the time of the index medical consultation for a minor fracture (index date) was measured using the ERA index [25]. ERA includes multidimensional risk factors over the previous 2 years (social, psychological, biological, clinical, cognitive and environmental components) [25]. The ERA index assigns specific weights to age, social components (marital status, race), physical components (history of diabetes, coronary artery disease, myocardial infarction, congestive heart failure, stroke, chronic obstructive pulmonary disease, cancer), cognitive components (history of cognitive impairments and dementia) and to consequences of frailty (number of hospital admission days in the 2 years before the index date of the minor fracture). The ERA scores of our population related to each of these components are described in Table 1. Globally, the ERA index scores vary from − 7 (lowest risk) to 34 (highest risk). As in Crane et al. [25], ERA scores were collapsed into five categories in the current study: robust seniors (ERA ≤ − 1); well seniors (ERA [0–3]); well seniors with treated comorbidities (ERA [4–8]); pre-frail seniors (ERA [9–15]); frail seniors (ERA ≥16). For implementation of the ERA index in the administrative databases, the marital status, which is a proxy for social support, was replaced by the social deprivation index quintiles that are routinely assigned in the QICDSS (see Covariates section below for details on this index). These quintiles were collapsed into three categories that were assigned weighted scores to align with the ERA: score − 1 (highest support: 1st and 2nd quintile), 0 (3rd quintile), + 1 (lowest support, 4th and 5th quintile). Race was not available, therefore not considered. The physical and cognitive components were considered as part of the index if there was one hospitalization or two physician billing claims associated to them and recorded at least 30 days apart, in the previous 5 years of the index date, excluding the 30 days prior to the index date [28]. ICD-9-CM and ICD-10-CA codes are described in Additional file 1: Table S1 and were used for identification of each physical or cognitive component.

**Table 1:** Elders risk assessment index

Crane et al. (2010)		Fillion et al. (2018)	
Parameters	Score	Parameters	Score
Married	-1	Social deprivation index	
Age		Highest support	-1
70–79	1	Average support	0
80–89	3	Lowest support	1
≥90	7	Age	
Race		65–69	0
Black	6	70–79	1
Other	0	80–89	3
Unknown	−6	≥90	7
Days in hospital during the previous 2 years before the index fracture		Days in hospital during the previous 2 years before the index fracture	
1–5	5	1–5	5
≥6	11	≥6	11
Medical history		Medical history	
Diabetes history	2	Diabetes history	2
History of CAD/MI/CHF	3	History of CAD/MI/CHF	3
History of stroke	2	History of stroke	2
History of COPD	5	History of COPD	5
History of cancer	1	History of cancer	1
Histort of dementia	3	Histort of dementia	3

*CAD* Coronary artery disease, *MI* Myocardial infarction, *CHF* Congestive Heart Failure, *COPD* Chronic obstructive pulmonary disease

### Covariates

#### Material and social deprivation index

Since it lacks individual socioeconomic information, the QICDSS routinely incorporates the material and social deprivation index, which is an ecological substitute of the socioeconomic status developed at INSPQ [26, 29] based on indicators from the Canadian Census. It combines information on education level, employment, average personal income, marital status, the proportion of people living alone, and single-parent families [26, 29]. This information is aggregated into two sets of quintiles (1: least deprived, 5: most deprived): material and social deprivation.

#### Number of comorbidities

The coding criteria developed by *Quan* et al. [30] was used to define 28 relevant Elixhauser comorbidities in the QICDSS database (Additional file 1: Table S2). As for the ERA index, an individual was considered to have a specific comorbidity if there was one hospitalization or two physician claims recorded at least 30 days apart, in the previous 5 years of the index date, excluding the 30 days prior to the index date [28]. The numbers of comorbidities (range from 0 to 28) were aggregated into three categories: 0–1, 2–4, and ≥ 5 comorbidities.

#### Anatomical site of fracture

To identify the incident non-hip fractures and sites, a validated algorithm was used [27]. The algorithm is designed to first select all medical services billing codes potentially associated with fracture treatment: (1) claims with medical services billing codes definitively related to fracture care or (2) claims with medical service billing codes not limited to fracture care. Fracture sites were defined by the specific medical service codes of the index claim related to the treatment of fracture or to the ICD-9-CM diagnostic codes. Finally, a 6-month period was considered as a “washout period” between two clinical sequences related to the same fracture to reduce potential misclassification of fracture follow-up as a new incident fracture. A sensitivity of 80% and a positive predictive value of 80% have been demonstrated for all fractures (except for vertebral fractures with sensitivity of 40%) [31]. The algorithm used to identify fracture in this study was typically develop and validated to identify fragility fracture (i.e. fracture occurring at an anatomical site recognized to be related to osteoporosis). Hand, craniofacial and toe fractures are not related to osteoporosis and was considered as traumatic fracture. Therefore, they were not considered by the algorithm [27].

#### Area of residence (rural/urban)

The Quebec geographical area is divided into 4 categories based on census data: Montreal census metropolitan (> 1,000,000 inhabitants), other census metropolitan (100,000 to 1,000,000 inhabitants), agglomerations (10,000 to 100,000 inhabitants) and rural (< 10,000 inhabitants) areas.

### Outcomes: Health services

Healthcare services use in the year prior and after the index date of the medical consultation for a minor fracture was measured for three distinct health services: emergency department (ED) visits, primary care practitioner (PCP) visits and hospitalizations. Because the ERA index includes days of hospitalizations in the 2 years before the index fracture, Hospitalization as an outcome was only measured in the year after the index fracture. These events were chosen as independent outcomes, as they are associated with premature institutionalization and high resource utilization [25, 32, 33]. Healthcare services use within ±7 days of the index fracture were excluded as they were considered to be directly associated to the trauma.

Using PCD, all medical services provided by an emergency specialist or in emergency care facilities were identified. The *number of emergency department (ED) visits* was computed according to the recommendations of *Belzile* et al. [34], which consider only one ED visit billing for two consecutive days of ED visits billing. Moreover, all ED visits billed during a hospitalization were excluded (i.e., ED visits between admission and discharge dates in Med-Echo).

Medical services with provider codes related to general practitioner and delivered in private care, outpatient or family medicine unit were selected to assess the *number of primary care practitioner (PCP) visits*. If a patient had seen a PCP several times or several PCP visits in two consecutive days, only one single visit to a PCP was considered.

Finally, in order to compute the *number of new hospital admissions after the index fracture,* hospital transfers were not considered as new admissions. At least 1 day between the previous discharge date and a new admission was required to consider a new episode. Hospital admissions with a vocation type related to rehabilitation, psychiatric or long-term care were not considered.

### Analyses

Characteristics of the study population were described using means and standard deviation (SD) for ordinal data and percentages for categorical data. The prevalence of frailty was estimated by the proportion of individuals assigned to the ERA ≥16 category. Mean, median and interquartile ranges were used to describe the health resource use in the year before and after the fracture according to five frailty categories based on the ERA scores.

Multivariate Generalized Estimating Equation (GEE) models were used to examine the relationship between frailty levels and health services while adjusting for covariates. In these models, Negative Binomial distributions were used with a period variable (before or after index date), the ERA index variable at baseline and their interaction. Since the number of hospital days before the fracture was considered in the establishment of the ERA index, the period variable was excluded in the model evaluating the association between frailty index and number of hospital days after the fracture. The models take into consideration the difference in the number of days that each patient is at risk of using health services (i.e., exclusion of in-hospital periods for ED and PCP visits outcomes and period after death for all three outcomes) by adding as a parameter an offset variable corresponding to the time of exposure. Covariables considered as potential confounding factors were age, sex, area of residence (rural/urban), site of fracture, number of comorbidities and material and social deprivation index. For all analyses, covariates were included in multivariate models if significant at a 5% alpha level. The possible collinearity between variables of the final model was verified using the condition index and the variance inflation factors.

Data were analyzed using the 9.4 version of the SAS statistical software.

## Results

The cohort consisted of 178,304 community-dwelling men and women aged 65 and over with non-hospitalized non-hip fractures. Their mean (SD) age was 75.5 (7.5) years and 74.2% were women (Table 2). More than half of the fractures were in upper limbs (wrist 20.0%, humerus 18.7%, elbow 12.3%). Fifty-two percent of the patients had two or more comorbidities at the index date.

**Table 2:** Characteristics of study cohort at index visit for a non-hip fracture according to frailty levels

Characteristics	Robust ERA ≤ −1	Well ERA [0:3]	Well/ comorbidities ERA [4:8]	Pre-frail ERA [9:15]	Frail ERA ≥ 16	Total
N (%)	9345 (5.2)	73,400 (41.2)	45,984 (25.8)	25,322 (14.2)	24,253 (13.6)	178,304
Sexe, %						
Women	69.6	75.6	75.0	72.0	72.0	74.2
Men	30.4	24.4	25.0	28.0	28.0	25.8
Age (Individual ERA index components), mean (SD)						
65 and over	66.9 (1.4)	72.7 (5.4)	78.3 (7.4)	77.9 (8.1)	79.7 (7.7)	75.5 (7.5)
Number of comorbidities, %						
0–1	85.3	69.8	43.0	18.5	4.0	47.5
2–4	14.6	29.0	49.8	57.2	37.3	38.8
≥ 5	0.1	1.2	7.2	24.2	58.7	13.8
Social deprivation index, % (Individual ERA index components)						
1 (more fortunate)	46.7	14.9	9.8	12.7	10.5	14.3
2	53.3	17.3	11.8	15.5	13.1	17.0
3	0.0	25.2	16.2	18.6	18.5	19.7
4	0.0	20.9	29.0	24.3	25.9	23.0
5 (less fortunate)	0.0	21.8	33.2	29.0	32.1	26.0
Material deprivation index, %						
1 (more fortunate)	17.9	19.1	19.0	17.6	15.8	18.3
2	18.5	18.4	18.4	17.8	17.8	18.2
3	19.4	20.1	19.7	20.3	20.5	20.1
4	20.8	21.1	21.9	21.5	22.1	21.5
5 (less fortunate)	23.5	21.3	21.0	22.8	23.8	21.9
Site of fracture, %						
Lower limbs	43.7	37.1	33.9	35.0	32.3	35.7
Upper limbs	49.9	55.4	55.6	52.1	51.2	54.1
Pelvis	1.8	2.6	3.9	4.7	5.9	3.6
Spine	4.6	4.9	6.6	8.2	10.6	6.6
Area of residence, %						
1 (Montreal CMA)	37.0	44.5	46.8	42.9	41.6	44.1
2 (Other CMAs)	16.9	18.5	19.6	18.9	19.7	18.9
3 (agglomerations)	11.2	13.5	14.8	15.6	17.5	14.6
4 (rural areas)	34.9	23.5	18.9	22.6	21.2	22.5
Physical and cognitive ERA, % (Individual ERA index components)						
Diabetes	0.0	8.6	22.3	23.6	34.5	17.3
CAD/MI/CHF	0.0	4.4	34.0	47.1	75.0	27.5
Stroke	0.0	1.1	6.8	13.6	25.1	7.5
COPD	0.0	0.0	10.4	26.5	51.3	13.4
Cancer	0.0	9.7	13.7	19.6	22.1	13.3
Dementia	0.0	0.2	3.8	7.0	14.8	4.1

*CMA* Census metropolitan area, *CAD* Coronary artery disease, *MI* Myocardial infarction, *CHF* Congestive Heart Failure, *COPD* Chronic obstructive pulmonary disease

**Fig. 2:**
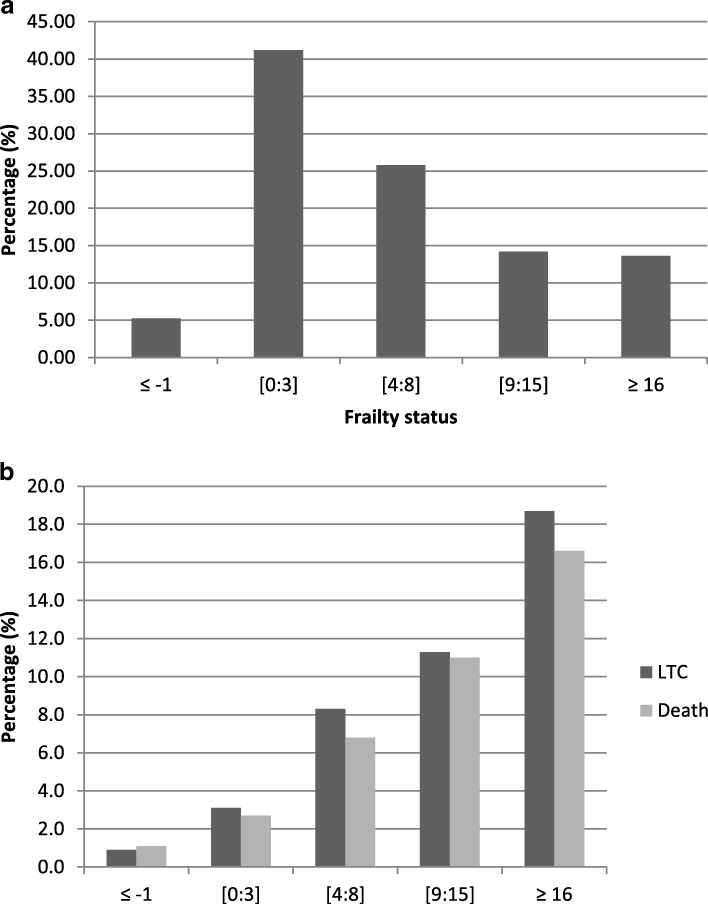
**a** Distribution of the study population according to frailty levels. **b** Distribution of admissions in long-term care (LTC) and death in the year post- fracture according to frailty levels

The ERA scores ranged from − 1 to 32. There were 13.6% (*N* = 24,253) frail seniors, while 5.2% (*N* = 9345) were considered as robust. The complete distribution of the study population along frailty levels is shown in Fig. 2a. Figure 2b shows marked increases in long term care admissions and deaths with frailty levels in the year post-fracture. Table 2 provides complete details on the cohort along the frailty levels. Briefly, the proportion of patients with 5 or more comorbidities increased with frailty levels from 0.1% for robust seniors to 58.7% for frail ones. Furthermore, 51.3% of frail seniors had a history of chronic obstructive pulmonary disease (COPD), 75.0% had a history of coronary artery disease (CAD), myocardial infarction (MI) or congestive heart failure (CHF) and 34.5% had a history of diabetes.

Overall, 64.7% of the frail seniors returned to ED and 27.9% were admitted to hospitals in the year following the fracture, while these proportions were significantly lower in robust individuals: 31.4 and 19.7%, respectively. Table 3 illustrates the mean number of ED and PCP visits as well as hospitalizations 1 year before and 1 year after the non-hip fracture according to frailty levels. For each type of service, there is a significant increase in health resource use with increased frailty levels. The multivariate regression analyses show that each increase in frailty levels is associated with a statistically significant increase in the adjusted risk for ED visits, both in the year before and after the fracture. For instance, compared to robust seniors, frail ones were more than four times more likely to visit the ED (Risk Ratio [RR]: 4.12; 95%CI: 3.74–4.55) in the year before sustaining their fracture and more than two times (RR = 2.69; 95%CI: 2.50–2.90) in the year post-fracture.

**Table 3:** Association between frailty and healthcare services use

Frailty (score)	Before index date			After index date			Interaction (frailty*Period)
	%	Mean (Median, Q1-Q3)	Adjusted RR	%	Mean (Median, Q1, Q3)	Adjusted RR	Adjusted RR
	Emergency Department (ED) visits						
Robust (ERA ≤ − 1)	19.9	0.32 (0, 0–0)	REF	31.4	0.55 (0, 0–1)	REF	1.80 (1.65–1.96)
Well (ERA 0:3)	23.3	0.37 (0, 0–0)	1.09 (1.00–1.20)	35.8	0.64 (0, 0–1)	1.20 (1.12–1.27)	1.96 (1.89–2.04)
Well/comorbidities (ERA 4:8)	32.8	0.55 (0, 0–1)	1.47 (1.35–1.61)	45.8	0.91 (0, 0–1)	1.63 (1.53–1.75)	1.99 (1.92–2.06)
Pre-frail (ERA 9:15)	54.4	1.11 (0, 0–2)	2.56 (2.33–2.81)	55.1	1.24 (1, 0–2)	2.06 (1.92–2.21)	1.44 (1.38–1.51)
Frail (ERA ≥16)	75.3	2.03 (1, 1–1)	4.12 (3.74–4.55)	64.7	1.70 (1, 0–2)	2.69 (2.50–2.90)	1.17 (1.13–1.22)
	Primary care practitioner (PCP) visits						
Robust (ERA ≤ − 1)	79.8	2.92 (2, 1–4)	REF^1^	81.8	3.19 (2, 1–4)	REF^1^	1.12 (1.09–1.14)
Well (ERA 0:3)	83.5	3.51 (3, 1–5)	1.14 (1.11–1.18)	84.3	3.69 (3, 1–5)	1.12 (1.09–1.16)	1.10 (1.09–1.11)
Well/comorbidities (ERA 4:8)	88.4	4.50 (4, 2–6)	1.33 (1.29–1.37)	85.1	4.38 (3, 1–6)	1.26 (1.22–1.30)	1.06 (1.05–1.07)
Pre-frail (ERA 9:15)	89.0	5.19 (4, 2–7)	1.43 (1.38–1.48)	82.3	4.54 (3, 1–6)	1.26 (1.22–1.30)	0.98 (0.97–1.00)
Frail (ERA ≥16)	88.9	5.87 (5, 2–8)	1.53 (1.47–1.59)	77.0	4.59 (3, 1–7)	1.28 (1.23–1.32)	0.93 (0.91–0.95)
	Number of hospital admissions						
Robust (ERA ≤ − 1)	4.8	0.06 (0, 0–0)	N/A	19.7	0.26 (0, 0–0)	REF	NA
Well (ERA 0:3)	6.9	0.09 (0, 0–0)	N/A	23.5	0.32 (0, 0–0)	1.26 (1.17–1.36)	NA
Well/comorbidities (ERA 4:8)	14.6	0.18 (0, 0–0)	N/A	45.8	0.44 (0, 0–1)	1.66 (1.53–1.80)	NA
Pre-frail (ERA 9:15)	43.0	0.58 (0, 0–1)	N/A	39.6	0.61 (0, 0–1)	1.96 (1.81–2.13)	NA
Frail (ERA ≥16)	67.0	1.16 (0, 0–2)	N/A	27.9	0.87 (0, 0–1)	2.34 (2.14–2.55)	NA
	Number of hospital days						
Robust (ERA ≤ − 1)		0.0 (0,0–0)	N/A		3.35 (0, 0–1)	REF	NA
Well (ERA 0:3)		0.0 (0, 0–0)	N/A		5.74 (0, 0–3)	2.15 (1.89–2.45)	NA
Well/comorbidities (ERA 4:8)		0.18 (0, 0–0)	N/A		10.46 (0, 0–10)	4.57 (4.00–5.22)	NA
Pre-frail (ERA 9:15)		4.22 (0, 0–4)	N/A		14.76 (2, 0–17)	5.48 (4.76–6.31)	NA
Frail (ERA ≥16)		12.54 (7, 0–17)	N/A		21.80 (9, 0–29)	7.57 (6.56–8.74)	NA

*RR* Risk Ratio, *REF* Reference category

Similarly, the risk of PCP visits was also significantly higher in each level of frailty, both before and after the fracture (Table 3). In frail seniors, the adjusted risk of PCP visits was 1.53 (95% CI: 1.47–1.59) in the year before the fracture and 1.28 (95% CI: 1.23–1.32) in the year post-fracture. In the year after the non-hip fracture, analyses also show a statistically significant increase in the risk of hospital admissions and hospital days with frailty levels. Indeed, compared to robust seniors, frail ones have an adjusted risk of 2.34 (95% CI: 2.14–2.55) for hospital admissions, and an adjusted risk of 7.57 (95% CI: 6.56–8.74) for the number of hospital days (Table 3).

Finally, Table 3 shows, for each level of frailty, the excess use of ED and PCP visits is potentially associated to the fracture in each frailty level. Compared to the pre-fracture year, our results suggest an almost twofold increase in the risk of ED visits in the post-fracture year for the first three frailty levels (RR_robust_ = 1.80 [95% CI:1.65–1.96], RR_well_ = 1.96 [95% CI: 1.89–2.04],

RR _well/comorbidities_ = 1.99 [95% CI: 1.92–2.06]). In pre-frail and frail seniors, the risk of ED visits increases respectively by 1.44 (95% CI: 1.38–1.51) and 1.17 (95% CI: 1.13–1.22) in the post-fracture period compared to the pre-fracture year. Finally, compared to the pre-fracture year, the risk of PCP visits in the post-fracture time only slightly increases among the robust, the well and the well/comorbidities groups.

## Discussion

In this study, we have tried to replicate the Elders Risk Assessment index (ERA index) using the QICDSS data. This index, which is based on a scoring system using information from community-dwelling elderly patients in administrative databases, was developed and validated by *Crane* et al [25]. This reproduction allowed us to identify frail patients at high risk of emergency department visits, general practitioner visits and hospitalizations in the year following a medical consultation for a non-hospitalized minor fracture. Several studies have developed frailty indexes but few have focused on the surveillance of these frail individuals at the population level [24].

We chose to reproduce the ERA index because it considers the multidimensional aspect of frailty and as it could be implemented in our specific provincial administrative data. Moreover, as we paralleled the inclusion/exclusion criteria of the clinical CETI cohorts in our database that covers virtually the whole population of the province of Quebec, our results reflect the actual use of health services by Quebec community-dwelling seniors with minor fractures. We used the QICDSS for our study, which is an innovative chronic disease surveillance system. It meets the five basic requirements of a public health surveillance system and it is based on health services use [26, 35–37]. Surveillance is important to measure the evolution of the health status of the population and the QICDSS is the most appropriate way to conduct chronic disease surveillance in Quebec [26]. Furthermore, the methodology used in this study and results obtained are certainly generalizable to other Canadian provinces who also have a similar universal healthcare system and medico-administrative databases. Moreover, aggregate data from QICDSS are transmitted on a regular basis to the Public Health Agency of Canada for their integration to the Canadian Chronic Disease Surveillance System for dissemination of surveillance product at Canadian level [26]. The methodology used can be exportable to other countries who used the International Classification of disease (ICD-9 and ICD-10) and similar medico-administrative databases to collect information on the management of their healthcare system. The results observed in our study are however dependent of the healthcare system organization and therefore probably not generalizable to other countries.

The potential pitfalls can be important in attempting to operationalize the complex phenomenon of frailty in data such as the QICDSS that are not initially designed for the surveillance of this specific health condition. However, our findings on frailty prevalence are consistent with the results obtained in the clinical CETI cohorts were 11.7% of community-dwelling seniors with minor fractures were found to be frail. Our results are also consistent with the systematic review conducted by Collard et al. [1]. They compiled the results on frailty prevalence of 21 different studies (with a total of 61,500 participants) and observed a frailty prevalence of 10.7% among seniors aged 65 and over (95% CI: 10.5–10.9) while we observed a frailty prevalence of 13.6%. Our slightly higher prevalence is most likely due to the nature of our population of older individuals who had sustained a fracture event.

Our results also concurred with other studies, including the ERA validation study [25]. In fact, *Crane* et al. included in their study 12,650 community-dwelling adults aged 60 and over. Patients were divided into five different groups and ERA scores ranged from − 7 to 32. 16.7% were in the most robust group while 9.4% were in the frailest group. This study identifies more robust people than ours, mainly because we selected a cohort of fractured elders and not on a general population of seniors. They also analyzed the number of emergency room visits and hospitalizations in the subsequent 2 years following an assignment to a primary care internal medicine provider. They found that compared to the lowest risk group, patients in the highest 10% risk group had a relative risk of 9.5 for either hospitalizations or ED visits (Odds Ratio [OR] = 9.5, 95% CI: 8.1–11.2), and an OR = 13.3 (95% CI: 11.2–15.9) for hospitalization alone over a 2 year period [25]. Furthermore, *Soong J.* et al. conducted a retrospective study to develop and validate a risk prediction model for acute care based on frailty syndromes [24]. The study used administrative data which included 2,099,252 patients over 65 years with ED admission to National Health Service in the UK [24]. They found that the frailty syndromes in addition to history of ED admissions demonstrated moderate discriminatory power, with the top 10% of patients at highest risk of ED readmissions within 30 days (39%) and being discharged to a higher level of support (17%) were at nearly twice the average population (ED: 21%, higher support: 9%) [24]. These findings are quite consistent with ours as we found that in the year following the fracture, more than twice frail seniors returned to ED compared to robust ones (64.7% vs 31.4%), and a larger proportion of them were hospitalized (27.9% vs 19.7%).

According to our knowledge, the current study is one of the first to attempt to measure the excess use of health services following a relatively minor fracture among frail elders. Our results suggest that special attention should be paid to elderly patients. The excess consumption of services is likely due to the fracture, which should therefore trigger additional assessment and care as soon as the patient first visits a health provider for a seemingly minor fracture. Indeed, it was shown in clinical cohorts that ED consultations for non-hospitalized injuries were associated with a marked decrease in quality of life [5] and an overall 16% rate of functional decline in the 6 months post-injury with frail seniors being at 10 times higher risk for such decline [18]. Our population-based statistical models also suggest that the increase in health services consumption is more important in seniors who are not frail yet compared to frail ones who were already high service users prior to their fracture. This concurs with the fact that in the frailest individuals, the consequences of a seemingly minor event (such as a minor fractures) are far more severe, triggering severe functional decline, which leads to long-term care placements and death (Fig. 2b) rather than more ED or PCP visits. Furthermore, the increase in health care use we observed in pre-frail seniors clearly supports the importance of addressing frailty in primary care [11, 38] (eg. ED and community clinics) in order to identify pre-frail seniors who are amenable to frailty preventive measures [39].

This study has limitations. First of all, even if we used validated algorithms for our analyses, the use of administrative databases may lead to possible omissions or coding errors. Coding data was used to identify comorbid conditions included in the ERA index. Coding data may under-estimate secondary diagnoses, however, other authors have found that administrative data such as ICD-9 codes typically correlate well with patient chart diagnoses [25, 30, 40].

Secondly, we could not perfectly replicate the ERA index. First, we used the social deprivation index instead of the simple marital status. The potential downside of this index is that it is not measured at the individual level. However, it based on the smallest census area units available in Canada and includes the proportions of widowed, of separated or divorced people, of people living alone and of single-parent families. We consider this to be a better variable to include in a frailty index since it contains more information about the strength of the social network of individuals. Moreover, this social deprivation index is widely used in surveillance and research activities as an important determinant of population health in Canada [29]. We also have not included the race of the individual. Because the Quebec population has a Caucasian population of over 89% and only 3% black population [41], the lack of ethnicity in the measurement of frailty likely has a limited impact.

Finally, the use of health administrative databases inevitably leads to a lack of clinical information. However, the results obtained in the databases are consistent with the cohort studies on similar issues [15, 17, 19, 42].

## Conclusions

Identification of people who are at an increased risk of adverse health outcomes is one of the reason to measure frailty [43]. This study suggests that seniors, identified as frail by the ERA index and who had relatively minor fractures, use more health services after the incident fracture. In a public health perspective, this study indicates that it might be possible to use Quebec’s administrative databases for surveillance on frailty and its consequences among seniors.

## Additional file

Additional file 1: **Table S1.** List of codes used for Elders Risk Assessment (ERA) index. **Table S2.** List of codes used for Elixhauser comorbidity index. (DOCX 19 kb)

## Abbreviations

CAD: Coronary artery disease
CCI: Canadian Classification of Interventions
CETI: Canadian Emergency Team Initiative
CHAMP: Concord Health and Ageing in Men Project
CHF: Congestive heart failure
CI: Confidence interval
COPD: Chronic obstructive pulmonary disease
ED: Emergency Department
ERA: Elders Risk Assessment
FIPA: Fichier d’inscription des personnes assurées
GEE: Generalized Estimating Eq.
ICD − 10-CA: International Classification of Diseases, 10th Revision, Canada
ICD: International Classification of Diseases
ICD-9-CM: International Classification of Diseases, 9th Revision, Clinical Modification
INSPQ: Institut national de santé publique du Québec
MED-ECHO: Maintenance et exploitation des données pour l’étude de la clientèle hospitalière
MI: Myocardial infarction
OR: Odds Ratio
OS: Orthopaedic surgeon
PCD: Physician-Billing claims
PCP: Primary Care Practitioner
QICDSS: Quebec Integrated Chronic Disease Surveillance System
RAMQ: Régie de l’assurance maladie du Québec
RR: Relative Risk
SD: Standard deviation
SHARE: Survey of Health Ageing and Retirement in Europe
