# Supplementary material for: Frailty and health services use among Quebec seniors with non-hip fractures: a population-based study using adminsitrative databases
Source: BMC Health Serv Res. 2019 Jan 25;19:70. doi: 10.1186/s12913-019-3865-z (PMC6347825; doi:10.1186/s12913-019-3865-z)
Supplement: Supplementary file 1 — Table S1. List of codes used for Elders Risk Assessment (ERA) index. Table S2. List of codes used for Elixhauser comorbidity index. (DOCX 19 kb) [file 12913_2019_3865_MOESM1_ESM.docx]

**Table S1. List of codes used for Elders Risk Assessment (ERA) index**

|  | **CIM-9** | **CIM-10** |
| --- | --- | --- |
| **Physical components** | | |
| Diabetes | 2500, 2501, 2502, 2503, 2504, 2505, 2506, 2507, 2508, 2509 | E100, E101, E109, E110, E111, E119, E120, E121, E129, E130, E131, E139, E140, E141, E149, E102, E103, E104, E105, E106, E107, E108, E112, E113, E114, E115, E116, E117, E118, E122, E123, E124, E125, E126, E127, E128, E132, E133, E134, E135, E136, E137, E138, E142, E143, E144, E145, E146, E147, E148 |
| Coronary artery disease | 410, 411, 412, 413, 414, 4292 | I20, I21, I22, I23, I24, I25 |
| Myocardial infarction | 4100, 4101, 4109, 4110, 4111, 4116, 4111, 4116, 4119, 4120, 4128, 4129 | I21, I22, I252 |
| Congestive heart failure | 39891, 40201, 40211, 40291, 40401, 40403, 40411, 40413, 40491, 40493, 4254, 4255, 4256, 4257, 4258, 4259, 428 | I099, I110, I130, I132, I255, I420, I425, I426, I427, I428, I429, I43, I50, P290 |
| Stroke | 430, 431, 432, 433, 434, 435, 436, 437, 438 | I6 |
| Chronic obstructive pulmonary disease | 491, 492, 496, 5064 | J41, J42, J43, J44 |
| Cancer | 14, 15, 16, 161, 162, 163, 170, 171, 172, 174, 175, 176, 177, 178, 179, 18, 190, 191, 192, 193, 194, 195, 196, 197, 198, 199, 200, 201, 202, 2030, 2386 | C0, C1, C20, C21, C22, C23, C24, C25, C26, C30, C31, C32, C33, C34, C37, C38, C39, C40, C41, C43, C45, C46, C47, C48, C49, C50, C51, C52, C53, C54, C55, C56, C57, C58, C6, C70, C71, C72, C73, C74, C75, C76, C77, C78, C79, C80, C81, C82, C83, C84, C85, C88, C96, C97, C900, C902 |
| **Cognitive component** | | |
| Dementia | 290, 2941, 3312 | F00, F01, F02, F03, F051, G30, G311 |

**Table S2. List of codes used for Elixhauser comorbidity index**

|  | **CIM-9** | **CIM-10** |
| --- | --- | --- |
| **Heart failure** | 39891, 40201, 40211, 40291, 40401, 40403, 40411, 40413, 40491, 40493, 4254, 4255, 4256, 4257, 4258, 4259, 428 | I099, I110, I130, I132, I255, I420, I425, I426, I427, I428, I429, I43, I50, P290 |
| **Cardiac arrhythmias** | 4260, 42613, 4267, 4269, 42610, 42612, 4270, 4271, 4272, 4273, 4274, 4276, 4277, 4278, 4279, 7850, 99601, 99604, V450, V533 | I441, I442, I443, I456, I459, I47, I48, I49, R000, R001, R008, T821, Z450, Z950 |
| **Valvular disease** | 0932, 394, 395, 396, 397, 424, 7463, 7464, 7465, 7466, V422, V433 | A520, I05, I06, I07, I08, I091, I098, I34, I35, I36, I37, I38, I39, Q230, Q231, Q232, Q233, Z952, Z953, Z954 |
| **Pulmonary circulation disorders** | 4150, 4151, 416, 4170, 4178, 4179 | I26, I27, I280, I288, I289 |
| **Peripheral vascular disorders** | 0930, 4373, 440, 441, 4431, 4432, 4433, 4434, 4435, 4436, 4437, 4438, 4439, 4471, 5571, 5579, V434 | I70, I71, I731, I738, I739, I771, I790, I792, K551, K558, K559, Z958, Z959 |
| **Hypertension, uncomplicated** | 401 | I10 |
| **Hypertension, complicated** | 402, 403, 404, 405 | I11, I12, I13, I15 |
| **Paralysis** | 3341, 342, 343, 3440, 3441, 3442, 3443, 3444, 3445, 3446, 3449 | G041, G114, G801, G802, G81, G82, G830, G831, G832, G833, G834, G839 |
| **Other neurological disorders** | 3319, 3320, 3321, 3334, 3335, 33392, 334, 335, 3362, 340, 341, 345, 3481, 3483, 7803, 7843 | G10, G11, G12, G13, G20, G21, G22, G254, G255, G312, G318, G319, G32, G35, G36, G37, G40, G41, G931, G934, R470, R56 |
| **Chronic pulmonary disease** | 4168, 4169, 49, 500, 501, 502, 503, 504, 505, 5064, 5081, 5088 | I278, I279, J40, J41, J42, J43, J44, J45, J46, J47, J60, J61, J62, J63, J64, J65, J66, J67, J684, J701, J703 |
| **Diabetes, uncomplicated** | 2500, 2501, 2502, 2503 | E100, E101, E109, E110, E111, E119, E120, E121, E129, E130, E131, E139, E140, E141, E149 |
| **Diabetes, complicated** | 2504, 2505, 2506, 2507, 2508, 2509 | E102, E103, E104, E105, E106, E107, E108, E112, E113, E114, E115, E116, E117, E118, E122, E123, E124, E125, E126, E127, E128, E132, E133, E134, E135, E136, E137, E138, E142, E143, E144, E145, E146, E147, E148 |
| **Hypothyroidism** | 2409, 243, 244, 2461, 2468 | E00, E01, E02, E03, E890 |
| **Renal failure** | 40301, 40311, 40391, 40402, 40403, 40412, 40413, 40492, 40493, 585, 586, 5880, V420, V451, V56 | I120, I131, N18, N19, N250, Z490, Z491, Z492, Z940, Z992 |
| **Liver disease** | 07022, 07023, 07032, 07033, 07044, 07054, 0706, 0709, 4560, 4561, 4562, 570, 571, 5722, 5723, 5724, 5725, 5726, 5727, 5728, 5733, 5734, 5738, 5739, V427 | B18, I85, I864, I982, K70, K711, K713, K714, K715, K717, K72, K73, K74, K760, K762, K763, K764, K765, K766, K767, K768, K769, Z944 |
| **Peptic ulcer disease** | 5317, 5319, 5327, 5329, 5337, 5339, 5347, 5349 | K257, K259, K267, K269, K277, K279, K287, K289 |
| **AIDS** | 042, 043, 044 | B20, B21, B22, B24 |
| **Lymphoma** | 200, 201, 202, 2030, 2386 | C81, C82, C83, C84, C85, C88, C96, C900, C902 |
| **Metastic Cancer** | 196, 197, 198, 199 | C77, C78, C79, C80 |
| **Solid Tumor** | 14, 15, 16, 161, 162, 163, 170, 171, 172, 174, 175, 176, 177, 178, 179, 18, 190, 191, 192, 193, 194, 195 | C0, C1, C20, C21, C22, C23, C24, C25, C26, C30, C31, C32, C33, C34, C37, C38, C39, C40, C41, C43, C45, C46, C47, C48, C49, C50, C51, C52, C53, C54, C55, C56, C57, C58, C6, C70, C71, C72, C73, C74, C75, C76, C97 |
| **Rheumatoid Arthritis** | 446, 7010, 7100, 7101, 7102, 7103, 7104, 7108, 7109, 7112, 714, 7193, 720, 725, 7285, 72889, 72930 | L940, L941, L943, M05, M06, M08, M120, M123, M30, M310, M311, M312, M313, M32, M33, M34, M35, M45, M461, M468, M469 |
| **Coagulopathy** | 286, 2871, 2873, 2874, 2875 | D65, D66, D67, D68, D691, D693, D694, D695, D696 |
| **Weight loss** | 260, 261, 262, 263, 7832, 7994 | E40, E41, E42, E43, E44, E45, E46, R634, R64 |
| **Fluid Electrolyte Disorders** | 2536, 276 | E222, E86, E87 |
| **Blood loss + Deficiency Anemia** | 2800, 2801, 2802, 2803, 2804, 2805, 2806, 2807, 2808, 2809, 281 | D500, D508, D509, D51, D52, D53 |
| **Alcohol abuse** | 2652, 2911, 2912, 2913, 2915, 2916, 2917, 2918, 2919, 3030, 3039, 3050, 3575, 4255, 5353, 5710, 5711, 5712, 5713, 980, V113 | F10, E52, G621, I426, K292, K700, K703, K709, T51, Z502, Z714, Z721 |
| **Drug abuse** | 292, 304, 3052, 3053, 3054, 3055, 3056, 3057, 3058, 3059, V6542 | F11, F12, F13, F14, F15, F16, F18, F19, Z715, Z722 |
| **Psychoses** | 2938, 295, 29604, 29614, 29644, 29654, 297, 298 | F20, F22, F23, F24, F25, F28, F29, F302, F312, F315 |
| **Depression** | 2962, 2963, 2965, 3004, 309, 311 | F204, F313, F314, F315, F32, F33, F341, F412, F432 |
| **Dementia** | 290, 2941, 3312 | F00, F01, F02, F03, F051, G30, G311 |
| **Osteoporosis** | 7330, 7331 | M80, M81, M82 |
